# Supplementary material for: Is Less Sedentary Behavior, More Physical Activity, or Higher Fitness Associated with Sleep Quality? A Cross-Sectional Study in Singapore
Source: Int J Environ Res Public Health. 2020 Feb 19;17(4):1337. doi: 10.3390/ijerph17041337 (PMC7068518; doi:10.3390/ijerph17041337)
Supplement: Supplementary file 1 [file ijerph-17-01337-s001.pdf]

S1 and S2

Table S1. Descriptive Statistics of Key Outcome Variables (N = 757).

	**Total**	Pittsburgh sleep quality index (PSQI) [a]		P-value
		Good sleep quality	Poor sleep quality	
**Accelerometer data**				
Average wear days	6.77 (0.64)	6.78 (0.64)	6.74 (0.63)	.581
Average wear time (hrs/day)	14.23 (1.44)	14.23 (1.43)	14.19 (1.52)	.787
MVPA (min/day) [b]	45.79 (21.11)	46.19 (21.1)	43.16 (21.1)	.182
Lower (1st and 2nd tertiles)	-	433 (65.9%)	72 (72.0%)	
Higher (3rd tertile)	-	224 (34.1%)	28 (28.0%)	
SB (hrs/day) [c]	7.86 (1.25)	7.84 (1.25)	8.04 (1.22)	.141
Lower (1st and 2nd tertiles)	-	440 (67.0%)	64 (64.0%)	
Higher (3rd tertile)	-	217 (33.0%)	33 (33.0%)	
PSQI components [d]				
Duration of sleep	0.65 (0.76)	0.52 (0.65)	1.48 (0.9)	<.001
Sleep disturbance	0.97 (0.48)	0.91 (0.45)	1.37 (0.51)	<.001
Sleep latency	0.77 (0.84)	0.61 (0.71)	1.84 (0.9)	<.001
Sleepiness	0.73 (0.67)	0.63 (0.61)	1.37 (0.72)	<.001
Sleep efficiency	0.02 (0.22)	0 (0.04)	0.12 (0.59)	<.001
Sleep quality	0.13 (0.36)	0.05 (0.22)	0.62 (0.6)	<.001
Medications to sleep	0.02 (0.17)	0 (0.04)	0.14 (0.43)	<.001
Total PSQI scores	3.29 (1.92)	2.73 (1.28)	6.94 (1.34)	<.001
Cardiorespiratory fitness (CRF)				
$MaxVO_2$ [e]	35.39 (4.56)	35.64 (4.5)	33.78 (4.68)	<.001
Lower (1st and 2nd tertiles)	-	424 (64.5%)	80 (80.0%)	
Higher (3rd tertile)	-	233 (35.5%)	20 (20.0%)	

MVPA = moderate-to-vigorous intensity physical activity. SB= sedentary behavior. Values are presented as mean and standard deviation unless otherwise described. LM represents the low-to-middle (1st and 2nd tertile) group. $MaxVO_2$ represents the estimated CRF. ST represents the sedentary time. [a] the categorization of participants into good and poor sleep quality was based on total scores of the PSQI (i.e., ≤5 and >5 for good and poor sleep quality, respectively). [b] The tertile groups of MVPA (min/day) were created based on cut-points of <35.65, <52.12, and ≥52.12 min/day for the 1st, 2nd, and 3rd tertiles. [c] The tertile groups of ST (hrs/day) were created based on cut-points of <7.50, <8.50, and ≥8.50 hrs/day for the 1st, 2nd, and 3rd tertiles. [d] The score range of PSQI components was from 0 (better) to 3 (worse) and total PSQI scores ranged from 0 (better) to 21 (worse). [e] The tertile groups of CRF were created based on cut-points of <33.30, <37.70, and ≥37.70 ml/kg/min for the 1st, 2nd, and 3rd tertiles.

Table S2. Correlations Between SB, MVPA and CRF.

**Pair of variables [a]**	***r* (95% CI)**
MVPA (min/day) & SB (hrs/day)	-0.30 (-0.36, -0.23)
MVPA (min/day) & CRF ($MaxVO_2$)	0.27 (0.20, 0.33)
SB (hrs/day) & CRF ($MaxVO_2$)	-0.04 (-0.11, 0.03)

MVPA = moderate-to-vigorous intensity physical activity. SB = sedentary activity. CRF = estimated cardiorespiratory fitness. [a] MVPA and SB minutes were least-square adjusted for accelerometer wear time.
